# Quantification of hip effusion-synovitis and its cross-sectional and longitudinal associations with hip pain, MRI findings and early radiographic hip OA

**DOI:** 10.1186/s12891-020-03532-7

**Published:** 2020-08-10

**Authors:** Harbeer Ahedi, Dawn Aitken, Leigh Blizzard, Flavia Cicuttini, Graeme Jones

**Affiliations:** 1 Menzies Institute for Medical Research, University of Tasmania, Hobart, Tasmania Australia; 2 Institute of Musculoskeletal Health, Sydney, NSW Australia; 3 Sydney School of Public Health, The University of Sydney Faculty of Medicine and Health, Sydney, New South Wales Australia; 4 Department of Epidemiology and Preventive Medicine, School of Public Health and Preventive Medicine, Monash University, Alfred Hospital, Melbourne, VIC 3004 Australia

**Keywords:** Musculoskeletal disorders, Osteoarthritis, Hip pain, Effusion-synovitis, Articular cartilage, Subchondral BMLs, Radiological hip OA

## Abstract

**Background:**

Hip effusion-synovitis may be relevant to osteoarthritis (OA) but is of uncertain etiology. The aim of this study was to describe the cross-sectional and longitudinal associations of hip effusion-synovitis with clinical and structural risk factors of OA in older adults.

**Methods:**

One hundred ninety-six subjects from the Tasmanian Older Adult Cohort (TASOAC) study with a right hip STIR (Short T1 Inversion Recovery) Magnetic Resonance Imaging (MRI) on two occasions were included. Hip effusion-synovitis CSA (cm^2^) was assessed quantitatively. Hip pain was determined by WOMAC (Western Ontario and McMaster Universities Osteoarthritis) while hip bone marrow lesions (BMLs), cartilage defects (femoral and/or acetabular) and high cartilage signal were assessed on MRI. Joint space narrowing (0–3) and osteophytes (0–3) were measured on x-ray using Altman’s atlas.

**Results:**

Of 196 subjects, 32% (*n* = 63) had no or a small hip effusion-synovitis while 68% (*n* = 133) subjects had a moderate or large hip effusion-synovitis. Both groups were similar but those with moderate or large hip effusion-synovitis were older, had higher BMI and more hip pain. Cross-sectionally, hip effusion-synovitis at multiple sites was associated with presence of hip pain [Prevalence ratio (PR):1.42 95%CI:1.05,1.93], but not with severity of hip pain. Furthermore, hip effusion-synovitis size associated with femoral defect (βeta:0.32 95%CI:0.08,0.56). Longitudinally, and incident hip cartilage defect (PR: 2.23 95%CI:1.00, 4.97) were associated with an increase in hip effusion-synovitis CSA. Furthermore, independent of presence of effusion-synovitis, hip BMLs predicted incident (PR: 1.62 95%CI: 1.13, 2.34) and worsening of hip cartilage defects (PR: 1.50 95%CI: 1.20, 1.86). While hip cartilage defect predicted incident (PR: 1.11 95%CI: 1.03, 1.20) and worsening hip BMLs (PR: 1.16 95%CI: 1.04, 1.30).

**Conclusions:**

Hip effusion-synovitis at multiple sites (presumably reflecting extent) may be associated with hip pain. Hip BMLs and hip cartilage defects are co-dependent and predict worsening hip effusion-synovitis, indicating causal pathways between defects, BMLs and effusion-synovitis.

## Background

Osteoarthritis (OA) is characterized by alterations in composition, structure and function of the various components of joint, including synovium [1–4]. OA has been historically categorized as non-inflammatory arthritis [2, 4]. However, synovitis plays a key role in cartilage damage and vice versa [1, 2, 5]. Occurring in either early or late stages of OA, synovitis leads to increase in catabolic and proinflammatory mediators such as cytokines, nitric oxide, prostaglandin E, and neuropeptides. These mediators produce excess proteolytic enzymes, which cause cartilage matrix degradation. In turn, cartilage breakdown leads to worsening synovitis [2, 6].

Several studies have reported associations of knee effusion-synovitis proving that it is one of the causes of knee pain, has an adverse effect on cartilage and is linked with radiographic knee OA [3, 7–10]. Although effusion-synovitis is a significant clinical prognostic factor for OA [11], at the hip it remains under-investigated.

A small retrospective study was the first to report hip effusion in twelve out of twelve subjects and severe synovitis in nine out of twelve subjects with hip RDOA (Rapidly Destructive OA) but did not demonstrate correlations of hip effusion or synovitis [12]. Subsequently, in a clinical study, hip effusion was reported in 70% of the subjects and major or/and asymmetrical hip effusion associated not only with hip pain but also with hip radiographic OA (ROA) [13]. In a study, Magnetic Resonance Imaging (MRI) was used for evaluating hip effusion and synovitis separately, a weak association between grade 1 synovitis (but not grade 2) and hip pain was found. However, in subjects with either synovitis or effusion, severe hip ROA was prevalent [14]. In a retrospective study, extensive synovitis was found in subjects with RDOA than in those with hip OA, indicating that higher synovitis could be related with rapid disease progression [15]. Modest correlations between hip effusion and hip ROA were demonstrated in a study designed to evaluate hip OA. However, no association between hip effusion-synovitis and hip pain score [16]. Interestingly, an MRI study conducted in athletics demonstrated lower prevalence of effusion-synovitis in hips with pain than without pain (Odds Ratio: 0.46 (95% CI: 0.3, 0.8) [17].

In a small study, investigating the effect of hyaluronic acid (HA) in injection in those with and without hip OA reported that hip pain was correlated with effusion-synovitis (*r* = 0.27, *p* = 0.03) and was lower in participants injected with HA (13.9 vs 7.8, *P* < 0.001) [18] Another study, showed that the frequency of effusion (*p* = 0.013) and reactive synovitis (*p* < 0.001) was greater in those with cam impingement [19].

Overall, the etiology of hip effusion-synovitis is underreported, and current data is inconclusive. Due to its proximity to the hip cartilage and other structures, hip effusion-synovitis could be a significant progenitor of hip OA. Effusion-synovitis could be a potential target for future clinical trials [20]. Hence, this study aims to describe the cross-sectional and longitudinal associations of hip effusion-synovitis in a large community-based sample.

## Methods

### Subjects

The Tasmanian Older Adult Cohort (TASOAC) study is an ongoing prospective, population-based study initiated in 2002 and has been extensively described in previous studies [21]. During the TASOAC study, a hip protocol was added during the latter part of phase 2. Hip MRI scans for phase2 and phase3 were conducted approximately 2.3 years apart. In the current study a sample of 245 consecutive participants who had a STIR (Short T1 Inversion Recovery) MRI sequence at phase 2 and/or phase 3 were included. Of these 245 participants, 30 participants were lost to follow-up in phase 3 and 17 subjects had no STIR MRI at phase 2. Of 198 subjects, hip effusion-synovitis could not be adequately assessed in the MRI scans of 2 subjects and these were excluded. Accordingly, a total of 196 subjects with complete data were included in this study. Written informed consent was obtained from all participants and the Southern Tasmanian Health and Medical Human Research Ethics Committee approved this study (approval number: H6488).

### Clinical and hip pain measures

Height, weight, Body mass index (BMI) were measured using standard protocols. Hip pain was determined using a hip specific Western Ontario and McMaster Universities osteoarthritis (WOMAC) index pain score. WOMAC uses a ten-point scale from 0 (indicating no pain) to 9 (indicating severe pain). Hip pain (five items) was assessed using the following questions: ‘Referring to your hips only, how much pain did you experience when walking on flat surface, going up and down the stairs, at night while in bed, sitting or lying, and standing upright.’ These five items were summed to create a total hip pain score, each with a possible range from 0 to 45 [22].

### Magnetic resonance imaging (MRI) assessment

For those with a hip MRI, the right hip was imaged in the sagittal plane using a 1.5 Telsa G. E signal whole-body magnetic resonance unit with a phased-array flex coil. Two MRI sequences were conducted for each participant. Sagittal images were obtained at a partition thickness of 1.5 mm with an in-plane resolution of 0.39 × 0.39 mm (512 × 512 pixels) using, a T1-weighted, fat-suppressed, 3-dimensional gradient-recalled acquisition in the steady state. The parameters for this were: flip angle 55 degrees; repetition time 58 ms; echo time 12 ms; inversion time (IT) 130 ms; field of view 16 cm; 60 partitions; 512 × 512–pixel matrix; acquisition time 11 mins 56 s, and one acquisition [23]. A second set of sagittal images was obtained with a slice thickness of 3.5 mm and an inter-slice gap of 1.5 mm using a STIR-weighted, fat saturation two-dimensional fast spin echo sequence. This sequence used a repetition time 4340 ms, echo time 28.4 ms; field of view 20 cm; 15 partitions (16 slices) and 512 × 512 pixel matrix [24]. All MRI measures were conducted on STIR MRI sequence using OsiriX imaging software (University of Geneva, Geneva, Switzerland).

### Quantitative assessment of hip effusion-synovitis

**Fig. 1:**
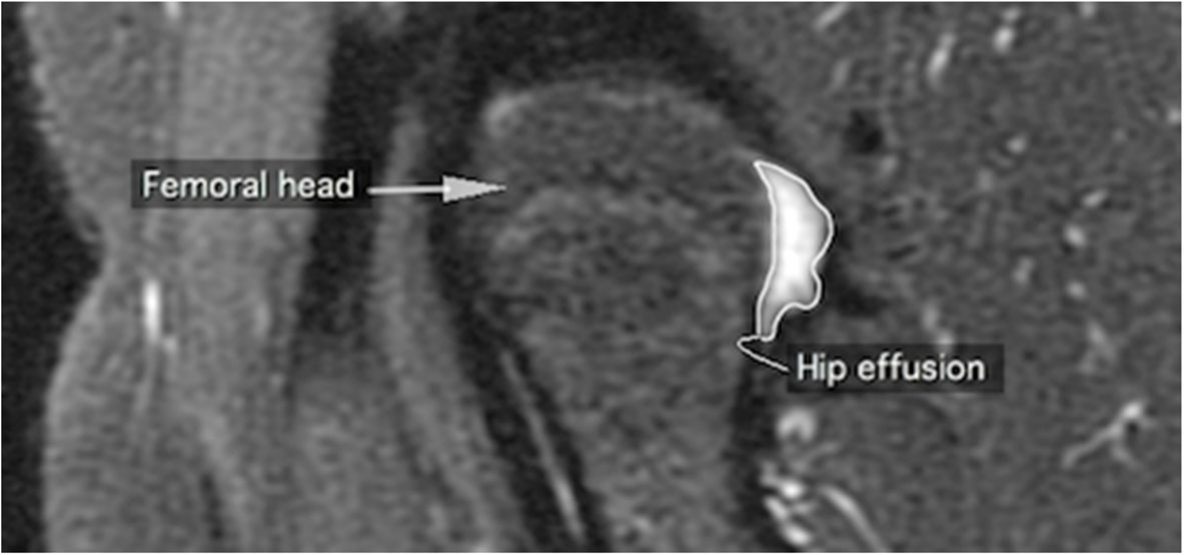
Assessment of hip effusion-synovitis using OsiriX imaging software

For quantitative measurements of hip effusion-synovitis, the observer (HA) selected the MRI slice with the largest effusion-synovitis and measured the maximum cross-sectional area (CSA) by drawing contours around the outer edges (Fig. 1). If the effusion-synovitis was present at more than one site around the femoral head (anterior, posterior or both), then the largest CSA of effusion-synovitis on each site was assessed. The reproducibility was evaluated in 40 subjects, with a 4 weeks interval between the two measures. The intra-rater agreement (kappa) for the presence of hip effusion-synovitis was 0.84, and the intra-class correlation coefficient (ICC) for hip effusion-synovitis CSA was 0.97. We have used similar method to assess knee hip effusion-synovitis [8] and also used these measurements in previously published studies [25, 26]. For analyses, the population was divided into two groups by median of effusion-synovitis CSA. The first group included subjects with no or small (< 0.77cm^2^) hip effusion-synovitis and the second group included subjects with moderate or large hip effusion-synovitis (≥0.77cm^2^).

### Assessment of hip cartilage defects, hip BMLs, and high cartilage signal

**Fig. 2:**
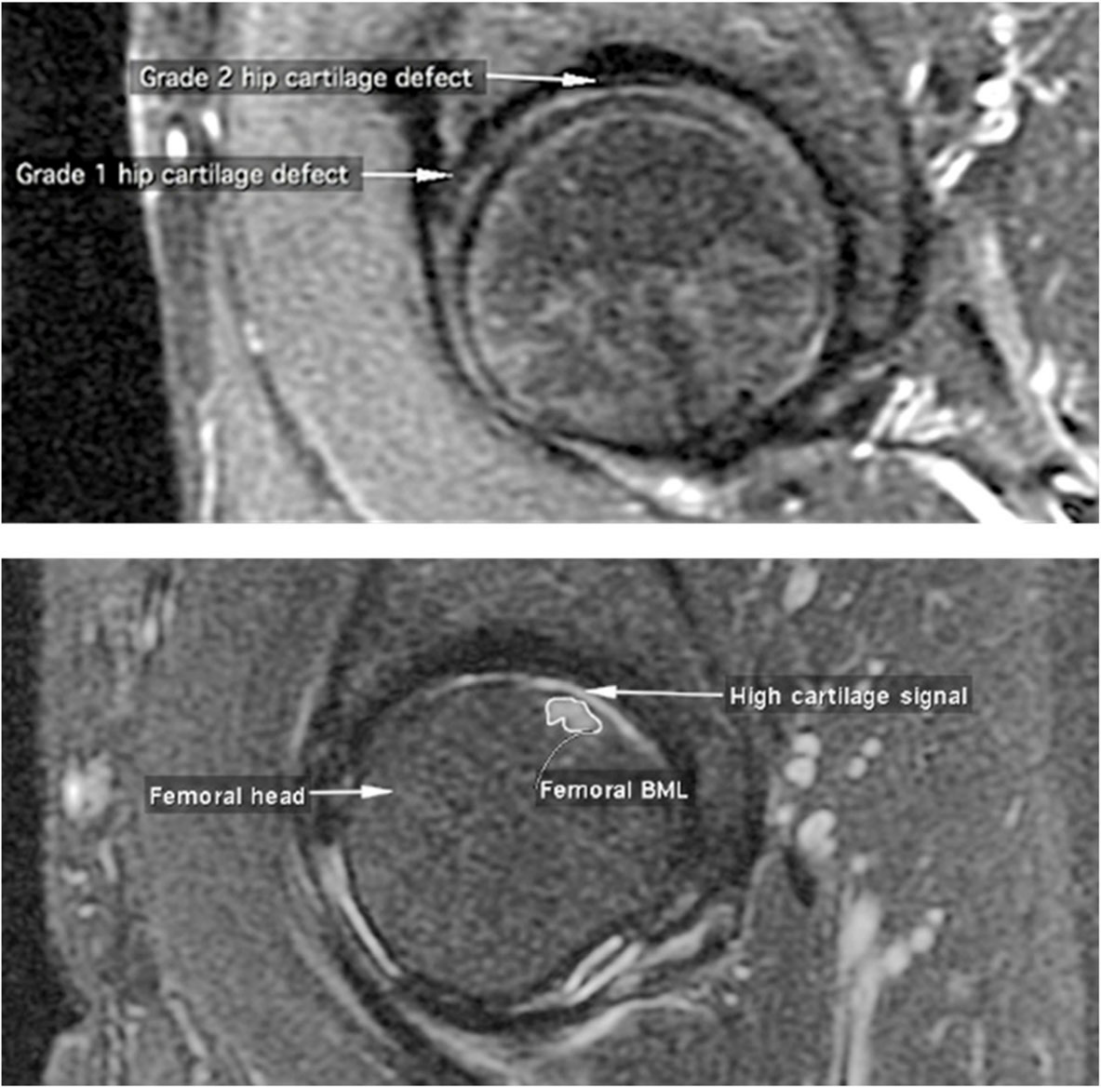
Measurement of hip BML, high cartilage signal and hip cartilage defects using OsiriX imaging software

Hip cartilage defects were assessed on MRI using OsiriX (Fig. 2). Hip defects on either femoral head or acetabulum were identified as any change in the hip cartilage and were categorized as; grade 0 = normal cartilage, grade1 = focal blistering or irregularities on the cartilage surface or a partial thickness defect and grade2 = full-thickness defect with bone ulceration and/or exposure of bone. If more than one defect was present at one site, the highest score was used. In a reliability study of 40 subjects with re-measurements after 4 weeks, the intra-rater agreement (kappa) was 0.89. Furthermore, the inter-rater reliability (kappa) assessed by two readers (*n* = 40) for the presence of cartilage defects and defect categories was 0.84 and 0.63 respectively [25].

Hip BMLs were identified as areas of increased signal intensity adjacent to the subchondral bone on the femoral head and/or the acetabulum. The observer manually selected the MRI slice with the largest BML and then determined the BML size (cm^2^) (Fig. 2). Intra-observer repeatability was assessed and the intra-class correlation coefficient (ICC) of the hip, femoral and acetabular BMLs was 0.98, 0.96 and 0.99 respectively [24].

High cartilage signal intensity change [27, 28] was defined as a high signal intensity band within the hip cartilage either adjacent to a hip BML or at any location on the STIR MRI slice if there was no BML present (Fig. 2). The intra-rater agreement (kappa) was 0.88 [24].

Methods for assessing hip cartilage defects, hip BMLs and cartilage were adapted by previously published grading systems used for assessment of structural changes in the knee detected by MRI [21, 29–32].

### Hip radiographs

Antero-posterior weight-bearing radiographs of the pelvis were obtained. Hip x-rays were read by two trained readers using the OARSI (Osteoarthritis Research Society international) grading system. The radiographic features of JSN and osteophytes of the right hip were graded on a 4-point scale, ranging from 0 to 3 where 0 = no disease and 3 = most severe disease by using an Altman’s atlas [33]. The intra-observer reliability for x-rays was carried out in 40 subjects and the ICC scores ranged from 0.60–0.87 [23, 34]. A non-zero score of either JSN or osteophytes was regarded as evidence of hip ROA. Thus, after combining JSN and osteophytes score, the presence of hip ROA was defined as a total score of 1 or greater.

### Statistical analysis

Ninety-four percent of the population had any hip effusion-synovitis. We were unable to differentiate between physiological (e.g. normal joint fluid) and pathological effusion-synovitis. Initial data-driven cut-off points did not reveal any significant results.,

Differences in demographical characteristics between participants who had no or small and moderate or large hip effusion-synovitis was calculated by using unpaired t-tests and chi-square tests (Table 1). Hip effusion-synovitis was also analyzed by the number of sites affected (independent of size) and continuously as CSA.

**Table 1:** Characteristics of the sample population

Characteristics	Small or no hip effusion-synovitis (*N* = 63)	Moderate or large hip effusion-synovitis (*N* = 133)	*P-value*
Age (yrs): mean (SD)	**63.4 (4.88)**	**64.6 (4.97)**	**0.04**
Male sex (%)	46% (29/63)	44% (58/133)	0.75
BMI (kg/cm^2^): mean (SD)	**27.2 (3.11)**	**28.0 (3.10)**	**0.02**
Hip pain			
Presence	**33% (19/63)**	**44% (58/133)**	**0.04**
Severity: mean (SD)	1.82 (5.41)	1.81 (5.45)	0.98
High cartilage signal	52% (33/63)	59% (77/133)	0.20
Presence of any bone marrow lesions (BMLs)	21% (13/63)	15% (20/133)	0.12
Hip cartilage defects			
Femoral defects	54% (34/63)	57% (75/133)	0.69
Acetabular defects	68% (42/63)	65% (86/133)	0.61
Any hip defects	71% (44/63)	71% (94/133)	0.92
Presence of radiographic hip OA (ROA)	44% (27/63)	50% (66/133)	0.59

The variable small or no hip effusion-synovitis includes participants with hip effusion-synovitis less than 0.77 cm^2^ and the variable moderate/large hip effusion includes participants with effusion-synovitis more than equal to 0.77 cm^2^

Data presented as means (SD) and proportions

Bold indicates statistically significant results (*p* < 0.05)

For cross-sectional analysis, log-binomial regression was employed to estimate the association between presence of hip pain, moderate/large hip effusion-synovitis and presence of hip effusion-synovitis at one or two/three sites. For estimating the relationship between severity of hip pain and categories of hip effusion-synovitis, linear regression of the logarithm of pain score on a binary covariate for hip effusion-synovitis was used. Log-binominal regression models were applied to investigate the associations between presence of hip BMLs, high cartilage signal, hip cartilage defects, hip ROA and presence of hip effusion-synovitis while linear regression was used to test the relationship between these factors and hip effusion-synovitis CSA. Associations between presence of hip BMLs and presence of cartilage defects were also investigated using similar models.

For longitudinal analyses, linear regression models were administered to estimate the relationship between change in hip pain and change in hip effusion-synovitis CSA. Similarly, the association between change in prevalence of hip BMLs, hip cartilage defects, hip ROA (baseline only) and change in hip effusion-synovitis CSA was examined using linear regression. Log-binominal regression was employed to investigate if hip BMLs predicted incident and worsening of cartilage defects and vice-versa (from phase 2 to phase 3). All results were presented as prevalence ratios (PR) and the models were adjusted for age, sex, body mass index (BMI), hip BMLs and hip cartilage defects as required. For cross-sectional analysis only, data on subjects at phase 2 and phase 3 were combined in analyses, and the correlation between repeated measurements on individuals was taken into account by adjusting standard errors using the sandwich (robust) estimator of variance [35, 36]. All statistical tests were two-sided and *p* values < 0.05 were considered significant and were conducted using Intercooled Stata 12 for Mac (Stata Corp, College station, TX, USA).

## Results

Overall, 196 participants from both phases were included in these analyses and the characteristics of the population is presented in Table 1. The population was split into two groups by the median of effusion-synovitis size. Participants with moderate/large effusion were older, heavier and mostly males. Presence of hip pain was more common in those with moderate/large hip effusion-synovitis, than in those with small or no hip effusion-synovitis. However, no differences were found in pain severity (hip pain> 0). For the imaging markers, of the two groups 20% of participants with small or no hip effusion-synovitis had a hip BML. Furthermore, presence of high cartilage signal and radiological hip OA was proportionate in both groups. Although those with moderate or large effusion-synovitis had more defects, no statistically significant differences were found.

Table 2 shows the cross-sectional associations between the presence and severity of hip pain and categories of hip effusion-synovitis. Overall, subjects with moderate/large hip effusion-synovitis had 31% greater hip pain but this association was not statistically significant. Nevertheless, those with hip effusion-synovitis at multiple sites had 42% higher hip pain in comparison to those with hip effusion- synovitis at only one site. Hip effusion- synovitis did not associate with severity of hip pain.

**Table 2:** Cross sectional associations between presence of hip pain and categories of hip effusion-synovitis

Study factor	Presence of hip pain	Severity of hip pain
Categories of hip effusion-synovitis	Adjusted PR (95%CI)^a^	Ratio of means (95%CI)^b^
Presence of moderate/large hip effusion-synovitis	1.31 (0.98, 1.74)	0.81 (0.53, 1.08)
Presence of hip effusion-synovitis at two/three sites	**1.42 (1.05, 1.93)**	0.99 (0.70, 1.40)

Independent variable: presence and severity of hip pain. Dependent variable: hip effusion-synovitis (moderate/large & multiple sites)

The variable small or no hip effusion-synovitis includes participants with hip effusion-synovitis less than 0.77 cm^2^ and the variable moderate/large hip effusion includes participants with effusion-synovitis more than equal to 0.77 cm^2^

^a^PR (95%CI) = prevalence ratios (95% confidence intervals) adjusted for age, sex, BMI, presence of hip BMLs, presence of cartilage defects and with clustering of observation on subjects at phase 2 and phase 3 taken into account

^b^Ratio of means (95% confidence intervals) adjusted for age, sex, BMI, presence of hip BMLs, presence of hip cartilage defects and with clustering of observation on subjects at phase 2 and phase 3 taken into account

Cross-sectionally, hip BMLs [(Prevalence Ratio (PR):0.75 95%CI:0.36,1.60)], high cartilage signal (PR:1.01 95%CI:0.85,1.21), hip cartilage defects (PR:1.12 95%CI:0.88,1.42) and hip ROA (PR:0.94 95%CI: 0.74,1.20) were not associated with the presence of hip effusion-synovitis. Nevertheless, any hip BMLs associated with any hip cartilage defects (PR: 1.22 95%CI 1.06, 1.40) independent of presence of hip effusion-synovitis. For these analyses; prevalence ratios (95% confidence intervals) were adjusted for age, sex, body mass index, presence of hip BMLs, presence of hip cartilage defects as required and clustering of observations on subjects at phase 2 and phase 3 was used using Huber-White estimator of variance.

Independent of presence of hip BMLs, hip effusion-synovitis associated with femoral cartilage defects (βeta: 0.32 95%CI 0.08, 0.56). No other structural or radiographic features of hip showed statistically significant associations.

Table 3 summarizes the longitudinal association between change in hip pain and change in hip effusion- synovitis CSA. Although, resolving hip effusion-synovitis showed a reduction in hip pain and worsening or persistent hip effusion- synovitis showed an increase in hip pain these analyses were not statistically significant.

**Table 3:** Longitudinal associations between change in presence of hip effusion-synovitis and change in hip pain from baseline to follow up

Study factor	n	Change in hip pain	Change in hip pain
		Adjusted *βeta (95% CI)*^a^	Further adjusted *βeta (95% CI)*^b^
No hip effusion-synovitis	8	Ref	Ref
Resolved hip effusion-synovitis	11	−0.20 (−1.51, 1.20)	− 0.31 (− 1.82, 1.20)
Worsening or persistent hip effusion-synovitis	174	+ 0.32 (− 0.81, 1.46)	+ 0.30 (− 0.91, 1.52)

Independent variable: change in hip pain. Dependent variable: change in hip effusion-synovitis CSA/size

n = number of observations in the analyses

Bold indicates statistically significant results

*CSA* cross-sectional area

^a^*βeta* co-efficient (95% confidence intervals) adjusted for age, sex and body mass index

^b^*βeta* co-efficient (95% confidence intervals) further adjusted for hip BMLs and hip cartilage defects at phase2

Table 4 outlines the longitudinal associations between change in effusion-synovitis, any hip BMLs and hip cartilage defects. Change in effusion-synovitis was not associated with BMLs but increased the risk of incident of hip cartilage defect by two-folds.

**Table 4:** Longitudinal associations between change in hip effusion-synovitis, any hip BMLs and cartilage defects

Study factor	Any hip BMLs			Hip Cartilage defects	
	Resolved	Incident	Persistent	Incident	Persistent
	Adjusted PR (95%CI)	Adjusted PR (95%CI)	Adjusted PR (95%CI)	Adjusted PR (95%CI)	Adjusted PR (95%CI)
Change in hip effusion-synovitis CSA					
Model 1	0.65 (0.30, 1.43)	1.31 (0.62, 2.75)	0.94 (0.46, 1.91)	**2.23 (1.01, 4.91)**	0.99 (0.88, 1.12)
Model 2	0.67 (0.32, 1.39)	1.25 (0.66, 2.33)	0.94 (0.49, 1.80)	**2.23 (1.00, 4.97)**	1.00 (0.89, 1.33)

Independent variable: presence of structural/ radiographic factors. Dependent variable: change in hip effusion-synovitis size

PR (95%CI) = prevalence ratios (95% confidence intervals)

Mode1 1 = prevalence ratios (95% confidence intervals) adjusted for age, sex, body mass index at phase 2

Mode1 2 = prevalence ratios (95% confidence intervals) adjusted for age, sex, body mass index, presence of hip BMLs, presence of cartilage defects at phase 2

Bold indicates statistically significant results

*CSA* cross-sectional area

Further longitudinal analyses demonstrated that any hip BML predicted incident (PR: 1.62 95%CI: 1.13, 2.34) and worsening hip cartilage defects (PR: 1.50 95%CI: 1.20, 1.86). Conversely, any hip cartilage defect predicted incident (PR: 1.11 95%CI: 1.03, 1.20) and worsening hip BMLs (PR: 1.16 95%CI: 1.04, 1.30). These analyses were independent of presence of hip effusion-synovitis.

## Discussion

This prospective cohort study describes the correlates of hip effusion-synovitis. Overall, there was no association between hip effusion-synovitis and hip pain. However, presence of hip effusion-synovitis at multiple sites (presumably reflecting effusion-synovitis extent) was associated with the presence of hip pain. Femoral defects were associated with hip effusion-synovitis CSA. High cartilage signal did not associate with hip effusion-synovitis and contradictory to previous research no relationship between hip ROA and hip effusion-synovitis was found. Change in effusion-synovitis increased the risk of hip cartilage defects.

Cross-sectionally, subjects with a hip effusion- synovitis at multiple sites were 42% more likely to have hip pain. At the hip, a few studies besides ours have reported these associations. The first study demonstrated that older adults with diagnosed hip OA with hip pain in mid-thigh and pain on palpation had higher odds of major hip effusion-synovitis [13]. While in the second study, a weak association was found between hip pain and grade1 but not grade2 synovitis [14]. The third found no correlation between hip effusion-synovitis and hip pain score [16]. Oddly, the fourth study showed higher prevalence of effusion-synovitis in athletics with lower hip pain [17] and the last study reported hip pain and effusion-synovitis were corelated.

These studies lack longitudinal data and measured effusion or synovitis semi-quantitatively. Also, only one of the above studies measured synovitis and effusion separately. While our study has its strengths, we did not assess site-specific hip pain and a cross-sectional association was found only in subjects who had extensive hip effusion-synovitis. We could not differentiate between physiological or pathological joint fluid but overall, it appears that site and extent of effusion-synovitis at the hip may be relevant for pain in OA.

Cross-sectionally, hip effusion-synovitis size was associated with femoral cartilage defects. Subsequently, in the longitudinal analyses, change in hip effusion-synovitis predicted incident of hip cartilage defects. An association between hip cartilage defects and hip effusion-synovitis has not been previously reported but most of the existing evidence for knee OA coincides with our findings. For instance, subjects with large knee effusion(>grade 2) at baseline had 2.7 times greater risk of cartilage damage at the end of 30 months follow-up [3]. A recent longitudinal study reported that regional knee effusion-synovitis predicted knee cartilage defects; cartilage volume loss and knee BMLs and these associations were largely mediated by cartilage defects [8]. Hence, causal pathways exist between knee effusion and knee cartilage defects; and suggests that knee cartilage defects could lead to the development of BMLs and cartilage volume loss at the knee joint [8]. Our results are comparable with these findings. Since there are limited number of studies on hip effusion-synovitis the nature of this study is mostly exploratory and further research is warranted.

Hip BMLs did not associate with hip effusion-synovitis. Nevertheless, independent of hip effusion-synovitis hip BMLs predicted worsening and incident of hip cartilage defects, and vice versa. Increase in hip effusion-synovitis increased the risk of developing cartilage defects.

We speculate that catabolic and proinflammatory mediators triggered by synovitis lead to intra-articular debris due to cartilage breakdown. In turn, presence of articular debris causes further inflammation of the synovium [1, 2, 4, 11]. Hypothetically, an increase in joint intra-capsular pressure could push synovitis into the subchondral bone through the cartilage defects causing the formation of BMLs. Moreover, BMLs are known to correlate with not only knee subchondral bone mineral density (BMD) [37] but also with local hip BMD [38, 39]. Thus, suggesting a causal pathway between hip effusion-synovitis, hip defects, hip BMLs and alterations in the bone itself [38, 39]. Moreover, effusion-synovitis is associated with hip shape [40] and studies show that those with cam impingement are more likely to have effusion-synovitis [19, 41].

### Limitations

We assessed hip effusion-synovitis quantitatively and have used similar methods previously [24, 42] and obtained high reproducibility. Nevertheless, the STIR MRI sequence used to examine joint effusion did not allow separation of physiological and pathological effusion and contrast-enhanced technique may yield clearer results. But our findings match with other MRI-based reports using similar techniques [13, 16]. Longitudinal analyses were carried out in a small number of subjects with hip BMLs. However, our findings were statistically significant and coincided with existing literature.

## Conclusions

Hip effusion-synovitis at multiple sites (presumably reflecting extent) may be associated with hip pain. Hip BMLs and hip cartilage defects are co-dependent and predict worsening hip effusion-synovitis, indicating causal pathways between defects, BMLs and effusion-synovitis.

Together, these factors have a deleterious effect on the bone and also contribute towards progression of OA.

## Data Availability

The datasets used and/or analyzed for the current study are available from the corresponding author.

## Abbreviations

BML: Bone marrow Lesions
BMI: Body Mass Index
ICC: Intraclass correlation coefficient
CI: Confidence interval
CSA: Cross sectional area
FAI: Femoroacetabular impingement
IT: Inversion time
KL: Kellgren-Lawrence
JSN: Joint space narrowing
OA: Osteoarthritis
MRI: Magnetic Resonance Imaging
OARSI: Osteoarthritis Research Society International
PR: Prevalence Ratios
RDOA: Rapidly destructive Osteoarthritis
ROA: Radiological Osteoarthritis
STIR: Short-TI Inversion Recovery
TASOAC: Tasmanian Older Adult cohort study
WOMAC: Western Ontario and McMaster Universities Osteoarthritis Index
